# Structure of RyR1 in native membranes

**DOI:** 10.15252/embr.201949891

**Published:** 2020-03-09

**Authors:** Wenbo Chen, Mikhail Kudryashev

**Affiliations:** ^1^ Max Planck Institute for Biophysics Frankfurt on Main Germany; ^2^ Buchmann Institute for Molecular Life Sciences Goethe University Frankfurt Frankfurt on Main Germany

**Keywords:** cryo‐electron tomography, muscle contraction, ryanodine receptor, sarcoplasmic reticulum, subtomogram averaging, Membrane & Intracellular Transport, Structural Biology

## Abstract

Ryanodine receptor 1 (RyR1) mediates excitation–contraction coupling by releasing Ca^2+^ from sarcoplasmic reticulum (SR) to the cytoplasm of skeletal muscle cells. RyR1 activation is regulated by several proteins from both the cytoplasm and lumen of the SR. Here, we report the structure of RyR1 from native SR membranes in closed and open states. Compared to the previously reported structures of purified RyR1, our structure reveals helix‐like densities traversing the bilayer approximately 5 nm from the RyR1 transmembrane domain and sarcoplasmic extensions linking RyR1 to a putative calsequestrin network. We document the primary conformation of RyR1 *in situ* and its structural variations. The activation of RyR1 is associated with changes in membrane curvature and movement in the sarcoplasmic extensions. Our results provide structural insight into the mechanism of RyR1 in its native environment.

## Graphical abstract

Ryanodine receptor 1 (RyR1) is a large Ca^2+^ release channel in the sarcoplasmic reticulum of skeletal and cardiac muscle. This study reports the structure of RyR1 in native membranes and reveals several features which have not been observed in the structures of purified RyR1s.

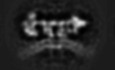

## Introduction

Ryanodine receptors (RyRs) are large ion channels performing Ca^2+^ release from the sarcoplasmic reticulum (SR) into the cytosol of skeletal and cardiac muscle, triggering muscle fibre contraction 1, 2. RyRs are homotetramers with a total molecular weight greater than 2.2 MDa that consist of a transmembrane domain forming an ion‐conductive pore, regulated by a large N‐terminal cytoplasmic domain 3, 4, 5. The cytoplasmic domain of RyR1, which is primarily expressed in skeletal muscle, interacts with Ca^2+^ and Mg^2+^ ions, ligands such as ATP, caffeine and ryanodine, and accessory proteins such as calmodulin (CaM) 6. CaM in its Ca^2+^‐unbound form is a weak agonist of RyR1, while in its Ca^2+^‐bound form it is an RyR1 antagonist. A 10‐kDa protein, S100A1, capable of increasing the open probability of RyR1, has been suggested to compete with CaM for the same binding site on the receptor 7. RyR1 has also been suggested to physically interact with the voltage‐gated Ca^2+^ channel (Ca_v_1.1), also known as the dihydropyridine receptor (DHPR), located in an invagination of muscle cell membrane called a transverse tubule (T‐tubule) 8. In the SR lumen, the major Ca^2+^‐buffering protein, calsequestrin (CSQ), interacts with RyR1 indirectly through the membrane‐anchored proteins triadin and junctin 9, each of which has a single transmembrane helix and a disordered intra‐SR domain. CSQ has two isoforms: CSQ1, which interacts with RyR1 in skeletal muscle, and CSQ2, which interacts with RyR2, a form primarily expressed in cardiac muscle 10. CSQ polymerizes in a Ca^2+^‐dependent manner 10, 11 and regulates the activity of RyR1 12. Biochemical analysis suggests that CSQ1 is the major protein component found in the sarcoplasmic reticulum at its junction with T‐tubules while along the longitudinal SR, the dominant protein is the 110‐kDa P‐type ATPase pump, sarco/endoplasmic reticulum Ca^2+^‐ATPase (SERCA) 13, 14. RyR gene mutations and the consequent dysfunction of RyR proteins have been linked to a number of pathologies, making RyRs an attractive drug discovery target 15.

Previous structural analyses of RyR1 have been performed by single‐particle negative stain and EM of purified receptors, typically in detergent 16, 17, or by a combination of EM maps with atomic models of RyR1 domains 18, 19, 20, 21. RyR1 has been visualized in native toadfish and zebrafish muscles 22, and a structure of the receptor from purified native muscle membranes was previously reported at a resolution of 71 Å 23. More recently, advances in single‐particle cryo‐EM 24 have led to sub‐5‐Å 25 and sub‐4‐Å 26 resolution structures of RyR1 in the apo state, as well as structures of RyR1 in nanodiscs in both closed and partially open states 27. Des Georges *et al* 28 determined a series of approximately 4‐Å reconstructions of RyR1 that proposed the following activation sequence: first Ca^2+^ or ATP “prime” the channel for opening, stabilizing the structure in a conformation permissive for opening; then a combination of three ligands, Ca^2+^, caffeine and ATP, open the channel. Alternatively, Ca^2+^ and ryanodine in combination lock the channel in an open state 28. Conformational changes from the ligand binding sites are transmitted to the pore several nanometres away via the central domain 29, and computational analysis suggests multiple routes by which ligand binding leads to channel activation [preprint: 30]. However, there are significant differences between the activation of RyR1 *in vitro* and *in vivo*. Transition between closed and open conformations of RyR1 is sensitive *in vitro* to applied detergents, which favour the closed state 31, and is generally modulated by a large number of small molecules 7, 12, which were only partially present with the purified receptors analysed by single‐particle cryo‐EM.

Here, we report a structure of RyR1 in native membranes purified from rabbit skeletal muscle, determined by cryo‐electron tomography and subtomogram averaging (StA). The structure includes the native membrane, shows observable curvature, as well as several interacting protein densities that were not observed in the reported high‐resolution cryo‐EM structures of purified RyR1. Our analysis reveals the most probable conformations of RyR1 *in situ* and decomposes its structural variation into principal components. Upon activation by Ca^2+^ and ryanodine, conformational changes in RyR1 lead to a noticeable change in the curvature of the membrane that potentially contributes to the energetics of channel opening and closing.

## Results and Discussion

### Structure of RyR1 in native SR membranes

**Figure 1:**
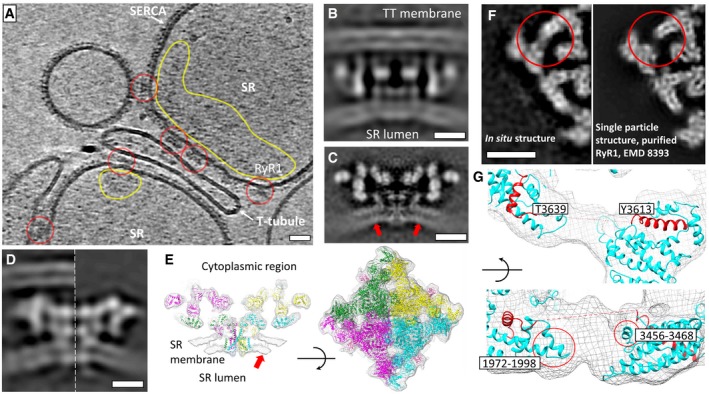
Structure of RyR1 in native SR membranes A slice through a filtered tomogram showing SR vesicles in contact with a putative T‐tubule. Individual RyR1 molecules are indicated by the red circles. The dense protein coat on the surface of the SR vesicles corresponds to the SERCA pump. Areas circled in yellow indicate accumulation of density inside the SR. Scale bar: 20 nm.A slice through the structure of RyR1 in contact with putative TT membrane.A middle slice through the *in situ* structure of RyR1 at 12.6‐Å resolution. The observed additional transmembrane density is indicated by the red arrows. Scale bars in B, C: 10 nm.Slice through the structures of apoRyR1 with the putative TT membrane (left) and the standalone apoRyR1 (right, with the flipped handedness and reduced resolution for comparison) 22 Å away from the middle slice (*Y* = 92 in the volume coordinates). Scale bar: 10 nm.Volume‐rendered visualizations of the average structure of RyR1 with the RyR1 atomic model fit (PDB: 5TB2). The left panel is a thin section through the centre of the volume.The location of the CaM/S100A1 binding site is marked with red circles in slices through maps of the *in situ* structure (left) and the single‐particle structure (EMDB: 8393) in the same orientation as the *in situ* structure and filtered to 15 Å (right). Scale bar: 10 nm.The *in situ* structure with an atomic model (PDB: 5TB2) fitted showing an unaccommodated density. Red circles are sites of potential interaction between RyR1 and the regulatory proteins.

We purified the SR‐containing fractions from rabbit muscle as previously described 32 and imaged them by cryo‐electron tomography. Similar to previously published work 23, SR vesicles were identified by their coating of small transmembrane proteins, thought to be SERCA (Fig 1A). RyR1 can be easily identified in both side and top views due to their characteristic shape and large size. Triad junctions composed of one T‐tubule flanked by two SR vesicles 32, 33 were observed in the tomograms. However, while some RyR1s were observed directly juxtaposed to the T‐tubule membranes, most were not. Furthermore, the SR membrane in the vicinity of each RyR1 was not smooth and had observable local curvature resulting in an undulating membrane appearance when several receptors were present (Fig 1A). Inside the SR lumen, higher protein density was observed in the vicinity of RyR1 as compared to other areas (Fig 1A).

**Figure EV1:**
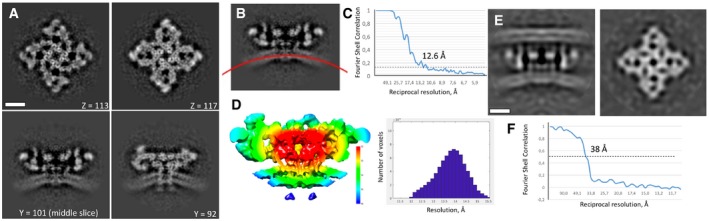
Structure of apoRyR1 *in situ* ASlices through the *in situ* average map or apoRyR1 in XY and XZ directions. Scale bar: 10 nm. Please note that due to observation through different section, the apparent curvature in the same structure may change. Therefore, it is critical to compare the curvature either at the same section or in sections traversing the middle of the particle.BMeasurement of the membrane curvature in the average structure.CResolution measurement of the structure in apo state by Fourier shell correlation between independently processed half‐maps.DLocal resolution of the structure with the corresponding colour code.E, FStructure of apoRyR1 in contact with the putative T‐tubule membrane and the corresponding resolution curve. Scale bars: 10 nm.

We used StA to determine the structure of RyR1 in native SR membranes. To ensure that RyR1 was in a closed state, we used EDTA to deplete Ca^2+^ from the sample. From the recorded tomograms, we manually picked 3,118 particles, out of which 205 had an observable adjacent T‐tubule‐like membrane density. From this subset, we generated an asymmetric reconstruction that showed C4 symmetry, which we applied for further refinement. The final structure had a resolution of 38 Å (Figs 1B and EV1). Interestingly, we could not detect an ordered density which could be attributed to DHPR, which we would expect to be distinguishable at such resolution. We further performed StA on all the available particles most of which were not juxtaposed to the putative T‐tubule membranes. An asymmetric reconstruction of a dataset consisting of the best 2,574 particles was C4 symmetric, and we therefore applied C4 symmetry for the subsequent alignment and reconstruction. As a consequence, all the features in the maps are C4 symmetric unless otherwise stated. The global resolution of the resulting structure is 12.6 Å (Figs 1C and EV1), with local resolutions ranging from 12 to 15.5 Å (Fig EV1). Local resolution is higher in the central domain, suggesting greater flexibility in the peripheral domains (Fig EV1). Overall, the structure resembles the reported cryo‐EM reconstructions of purified RyR1, particularly in its cytoplasmic domain (Fig EV1) 16, 17, 25, 26, 27, 28, 29, 34. The structures of standalone apoRyR1 and apoRyR1 adjacent to the putative T‐tubule membrane were very similar at the resolution of 38 Å with the peripheral domains positioned slightly further away from the SR membrane (Fig 1D).

**Figure EV2:**
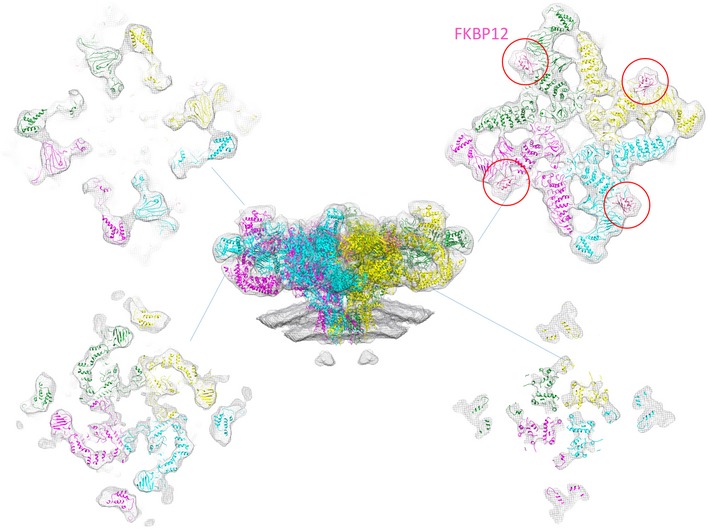
Rigid‐body docking of the atomic model 5TB2 to the *in situ* structure Slices through isosurface are presented perpendicular to the central panel at the heights indicated by the blue lines. FKBP12 is marked by red circles on the top right panel.

One of the previously reported structures of purified receptor in the apo state, EMD‐8393, one of multiple classes obtained at the time 28, is most similar to our *in situ* structure based on both Fourier shell correlation (Appendix Fig S1) and visual analysis of the rigid‐body fitting of the corresponding atomic model (PDB: 5TB2). This suggests that the captured conformation represents a closed apo state (apoRyR1) and we have therefore used the atomic model 5TB2 for further analysis (Fig 1E). Rigid‐body fitting of the atomic model revealed that the interaction sites of a protein called FK506‐binding protein 12 (FKBP12) are occupied (Fig EV2) and FKBP12 is bound to RyR1 and regulates its gating 35. As observed in the tomograms themselves, the StA structure also contains an SR membrane that is not flat, but instead follows the curvature of a sphere with a radius of approximately 50 nm (Fig EV1). By comparison, the diameters of the SR vesicles themselves were typically in the range of a few hundred nanometres. Closer examination of the average revealed a defined density visible approximately 5 nm from the edge of the transmembrane domain of RyR1 (Fig 1C). The density appears to connect the inner and outer leaflets. We suggest that this density corresponds to an ordered transmembrane helix; however, at the current resolution, we cannot conclude whether it is a single helix or a few closely positioned helices.

Our apoRyR1 map also shows density that, based on fitting of the atomic model, corresponds to residues 3,613–3,639 of the RyR1 protomer (Fig 1F). This density is not present in the reported high‐resolution cryo‐EM maps of isolated RyR1 (Fig 1F, Appendix Fig S1, Movie EV1), and consequently, it is not included in the existing atomic models of RyR1 (Fig 1F and C) 25, 26, 27, 28, 29, 34. The missing residues at this location have been previously shown to be the 17‐kDa CaM‐binding site 36 for which a 10‐kDa agonist S100A1 also competes 37. However, at the current resolution we cannot clearly identify the origin of the observed density. Alternatively, the density corresponding to the missing residues and the nearby lobes may be more ordered *in situ* compared to the structure of purified RyR1.

**Figure EV3:**
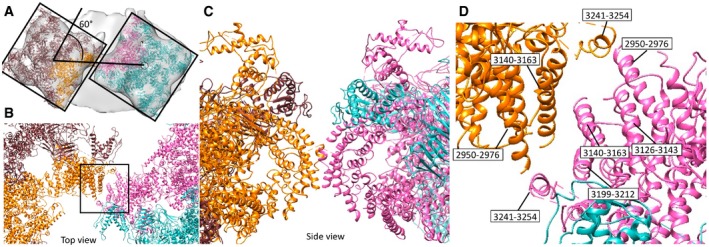
Inter‐protomer interactions between apoRyR1s in the paracrystalline array in native membranes AA subtomogram average map including two neighbouring apoRyR1s with two atomic models 5TB2 fitted.B, CTop and side views on the atomic models of the interacting domains.DZoom in to the area shown in (B) with the labelled interacting helices.

Previous studies using freeze‐fracture electron microscopy and cryo‐electron tomography report RyR1 forming a loosely ordered paracrystalline array 22, 23. Image classification revealed that out of 2,547 particles, 960 showed a neighbouring density. Of these, 242 resulted in the most ordered arrangement (Fig EV3). Rigid‐body fitting of the RyR1 atomic model (PDB: 5TB2) into the resulting density suggests that inter‐receptor interactions may be modulated by bridging solenoids. The closest contacts between the fit atomic models occur between corresponding helices 2,950–2,976, 3,126–3,143, 3,140–3,163, 3,199–3,212 and 3,241–3,254 of the neighbouring RyR1 tetramers (Fig EV3D).

### Interactions of RyR1 in the SR lumen

**Figure 2:**
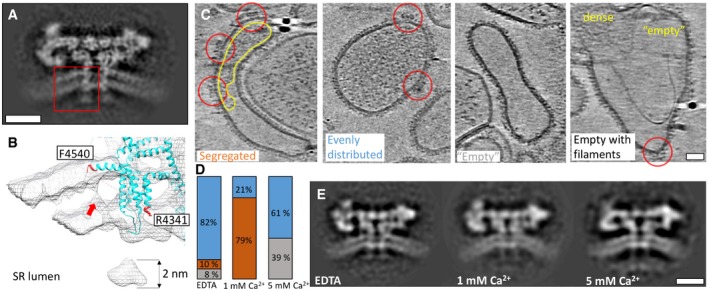
Intra‐SR extensions of RyR1 A slice through the average structure of the apo state at a position 22 Å away from the middle slice shown in Fig 1C. The intra‐SR extension is boxed on the density in red.An enlarged view of a volume‐rendered representation fitted with an atomic model (PDB: 5TB2) corresponding to the region boxed in (A). The red arrow points to a density between the bilayer leaflets; this density is at a different location from the one in Fig 1C and D.Representative SR vesicles with different distributions of inner SR density: segregated, evenly distributed, empty and empty with filaments. RyR1 particles are circled in red, and areas circled in yellow indicate accumulation of density inside the SR.The fractions of vesicles that fall into each density category for each RyR1 sample (EDTA: *n* = 64; 1 mM Ca^2+^: *n* = 32; 5 mM Ca^2+^: *n* = 21, technical replicates). Blue corresponds to the fraction of SR lumen vesicles showing evenly distributed density, orange to segregated density and grey to empty vesicles.Structures of RyR1 determined in the presence of increasing Ca^2+^ concentration (1 and 5 mM), all with intra‐SR extensions. Scale bars: 10 nm in (A) and (E), 20 nm in (C).

Inside the SR lumen, approximately 4‐nm long extensions originating from the transmembrane domain of RyR1 can be observed (Fig 2A and B). The local resolution of these extensions is lower than that of the cytoplasmic domain of RyR1, suggesting lower local order. Previously reported atomic models of purified RyR1 are missing the residues between R4341 in the cytoplasmic domain through to F4540 on the lumenal side. However, we are able to observe density traversing the inner and outer leaflet of the SR bilayer in the vicinity of F4540 (Fig 2B), adjacent to the transmembrane density presented in Fig 1C. Junctin and triadin both have single predicted transmembrane helices and long disordered intra‐SR domains. We were unable to identify distinct densities in the SR that could correspond to triadin and junctin; however, the ordered transmembrane density in the SR bilayer adjacent to the central pore could correspond to the transmembrane domains of triadin, junctin or both.

In previously published *in vitro* experiments, the Ca^2+^‐buffering protein CSQ1 located in the SR lumen was suggested to bind RyR1 via triadin and junctin at physiological Ca^2+^ concentrations (1 mM), and disassociate from RyR1 at Ca^2+^ concentrations that are either lower (≤ 1 mM) or higher (≥ 4 mM) than physiological level 10. We therefore probed whether the appearance of these density extensions could be manipulated by variation in intra‐SR calcium concentrations. To this end, we recorded tomograms of sample which had been depleted of Ca^2+^ (with 0.5 mM EDTA), were at physiological Ca^2+^ concentrations (1 mM) and were at high (5 mM) Ca^2+^ concentrations. In order to make the SR lumen accessible for Ca^2+^ supplementation, we added sub‐CMC concentrations of n‐Dodecyl‐B‐D‐Maltoside detergent (DDM) to the SR fraction to a final concentration of 0.005% (57% of the critical micelle concentration) in order to gently destabilize the SR membrane, and equalize the concentration of Ca^2+^ inside and outside the SR lumen. Tomographic data of RyR1 under each condition were collected. Segregation of protein density inside the SR lumen to regions adjacent to RyR1 was observed at physiological concentrations of Ca^2+^ in the presence of detergent (Fig 2C) and in our tomograms where no detergent had been added (Fig 1A) suggesting that the SR lumen has become accessible to Ca^2+^ after addition of detergent. Addition of detergent and depletion of calcium by EDTA changed the appearance of the density inside the SR from primarily “segregated” to primarily “evenly distributed” (Fig 2C and D). Addition of 5‐mM exogenous Ca^2+^ also resulted in either “evenly distributed” protein density or “empty” SR lumen (Fig 2C and D). We therefore speculate that the observed density in the SR lumen corresponds to CSQ1 which is reported to respond to changes in Ca^2+^ concentration 10. Filamentous densities inside and outside of the SR vesicles were observed in tomograms with 5 mM Ca^2+^(Fig 2C, rightmost panel). Some of these filaments observed inside the SR vesicles may be attributable to polymerized CSQ1, while those outside of the SR could be polymerized CSQ1 or actin.

Subtomogram averaging was performed on RyR1s for the varying Ca^2+^ concentrations, all of the resulting structures were similar and had intra‐SR extensions (Fig 2E). We therefore concluded that most of the density in the sarcoplasmic extensions does not respond to changes in calcium concentration. We suggest that the sarcoplasmic extension densities may represent some parts of residues 4,340–4,540 of RyR1, which are absent from the published atomic models due to higher flexibility in this region. We have updated the domain definitions based on the annotation by Des Georges *et al* 28 to include these sarcoplasmic extensions (Table 1). Interestingly, based on fitting of the atomic model, F4540 is located in the outer bilayer of the SR membrane, and A4350 in the inner bilayer, with no other transmembrane helices predicted in the interval between them by TMHMM 38. We therefore cannot currently estimate how many residues of RyR1 may be located in the SR lumen. Further structural analysis will be required to determine the composition of the sarcoplasmic extensions.

**Table 1:** Updated assignment of RyR1 residues into domains based on Des Georges *et al* 28

	Domain name	Abbreviation	Residues
1	N‐terminal domains A and B	AB domain	1–392
2	N‐terminal solenoid	Nsol	393–627
3	SP1a/ryanodine receptor domain 1	SPRY1	628–849
4	RYR repeats 1 and 2	Repeat 1–2	850–1,054
5	SP1a/ryanodine receptor domain 2	SPRY2	1,055–1,241
6	SP1a/ryanodine receptor domain 3	SPRY3	1,242–1,656
7	Junctional solenoid	Jsol	1,657–2,144
8	Bridging solenoid	Bsol	2,145–3,613
9	RYR repeats 3 and 4	Repeat 3–4	2,735–2,938
10	Shell‐core linker peptide, CaM and JSol binding sites	SCLP	3,614–3,666
11	Core solenoid	Csol	3,667–4,174
12	EF‐hand pair	EF1&2	4,060–4,134
13	Thumb and forefingers domain	TaF	4,175–4,253
14	Auxiliary transmembrane helices	TMx	4,322–4,370
15	Sarcoplasmic extensions	SExt	4,371–4,540
16	Pseudo‐voltage‐sensor domain	pVSD	4,541–4,819
17	Helical‐bundle domain between S2 and S3	S2S3	4,666–4,786
18	Channel pore domain	Pore	4,820–4,956
19	Cytoplasmic extension of S6	S6c	4,938–4,956
20	C‐terminal domain	CTD	4,957–5,037

### Structural variation of the RyR1 domains in native membranes

**Figure 3:**
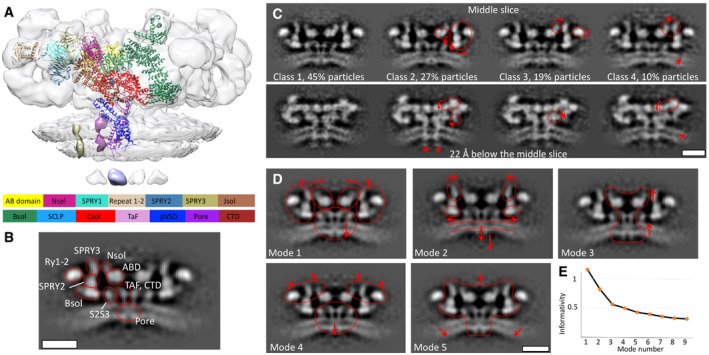
Structural heterogeneity of RyR1 *in situ* A, BThe domain definition displayed on the *in situ* structure, and the update from Ref 28 is presented in Table 1. A is a volume‐rendered representation with the atomic model (PDB: 5TB2), and B is domain representation on the greyscale image. Please note the location of two transmembrane densities: yellow—shown in Fig 1C and magenta—shown in Fig 3B.CSlices through the results of classification of the particles in the global average into 4 classes at resolutions 15.4, 18, 18 and 27 Å, respectively, with the percentages of the total particles in each class shown beneath. Red arrows show domain movements compared to class 1.DThe first five modes of correlated movements. Direction of motion is indicated by the arrows.EInformativity of the respective modes measured as variance of the corresponding mode divided by variance of the first mode. Scale bars: 10 nm.

We further aimed at analysing the structural heterogeneity of RyR1 domain movements in apo state in native membranes (Fig 3). Classification of the apoRyR1 particles into four distinct classes yielded one major class containing 45% of the particles, with the other classes less populated, with 27, 19 and 10% of the particles, respectively (Fig 3C and Appendix Fig S3). Sarcoplasmic extensions are apparent in all four classes (Fig 3C), suggesting that the majority of the particles have the corresponding density. Class 1 is most similar to the global average. The major differences between class 2 and class 1 are the downward movement of the AB domain (ABD) and the outward rotation of the peripheral domains. Comparing class 3 to class 1, in class 3 an outward movement of the N‐terminal solenoid towards the SPRY3 domain can be observed; this rotation is accompanied by a rotation of the Ry1‐2 domain. While classes 1–3 all had similar membrane curvature, the membrane curvature in class 4 is less pronounced, and class 4 also shows the Nsol and SPRY3 domains slightly extending in the cytoplasmic direction (Fig 3 and Movie EV1). At the current resolution, we could not determine reliable correlations between classification for the entire receptor and the classes obtained focusing on the region around 3,613–3,639. However, there was a clear difference in class occupancy when compared to the classifications performed by both des Georges *et al* 28 and Efremov *et al* 27 on purified RyR1. In the case of the single‐particle structures, the Ca^2+^‐depleted datasets had almost equal occupancy of their four classes. In contrast, the most populated class for our *in situ* data had approximately 45% of the particles, suggesting a different conformational spectrum from the purified RyR1. The most populated class *in situ* represented the global average and showed the highest similarity to class 2 from Des Georges *et al* (PDB: 5TB2), suggesting that this may be a more physiologically common conformation. The observed differences in the occupancy distribution across the classes may be due to the presence of curved native membrane, the occupancy of natively interacting and/or the presence of additional transmembrane proteins *in situ*.

In order to further understand the structural variation of RyR1 present in the data, we made use of principal component analysis inspired by Dashiti *et al* 39 and by Haselbach *et al* 40. Briefly, we performed multireference classification with alignment into a large number (50) of classes and then executed a principal component analysis on the result (see Materials and Methods). This analysis resulted, by definition, in multiple uncorrelated modes, ordered by the variance encapsulated within each of the modes. For example, mode 1 is defined as that encoding the highest variance and is therefore more informative than mode 5 (Fig 3D and E). Dividing the data into half‐sets along the eigenvector of each mode and comparing the resulting averages revealed the principal movements of apoRyR1 (Fig 3D, Appendix Fig S3, and Movies EV2, EV3 , EV4 , EV5 , EV6 and EV7):

*Mode 1*: global movement of the central domains in the direction of the membrane with an associated upward rotational movement of the peripheral domains of the receptor ([Link], [Link]). This mode also includes movement of the N‐terminal solenoid towards the SPRY3 domain suggesting the presence of two populations of RyR1 in native membranes with and without contact between the N‐terminal solenoid and the SPRY3 domain. It has been previously suggested that addition of a peptide corresponding to residues 590–628, which are located at the end of the N‐solenoid domain (Table 1), can cause enhancement of ryanodine binding to RyR1 and RyR2 41. In light of this, we hypothesize that mode 1 corresponds to two populations of RyR1 consisting of more and less active receptors. Interestingly, for mode 1, receptors with similar eigencoefficients, and therefore similar conformations, were also spatially located proximal to each other in the tomograms (Appendix Fig S4), suggesting that this conformational variation could be spatially regulated. The molecular mechanisms of such spatial regulation would need to be investigated further. Such a spatial relationship has not been observed for the other modes.
*Mode 2*: (71% of variance compared to the mode 1): an increase in the distance between the leaflets of the bilayer around the transmembrane domain of RyR1 is associated with minor movement of SPRY3 towards, and the bridging solenoid away, from the symmetry axis ([Link], [Link]). The variation in the distance between the leaflets of the bilayer could originate from compositional heterogeneity of bilayer itself, which would have implications for protein function.
*Mode 3*: (49% of variance compared to the mode 1): movement of the inner domains upwards along the central axis of the receptor coupled with a lower amplitude movement by the membrane in the same direction ([Link], [Link]).
*Mode 4*: (42% of variance compared to the mode 1): vertical stretch of the cytoplasmic domain of the receptor away from the membrane, coupled to displacement of the intra‐SR extensions away from the symmetry axis ([Link], [Link]). Modes 3 and 4 show two types of vertical elongation/contraction that may relate to a change in the distance between the SR membrane and the T‐tubules, something that can also occur *in vivo* as a response to membrane deformation. Similar conformational changes are present when comparing the apoRyR1 and apoRyR1 with the putative TT membrane structures (Fig 1D). However, the majority of our RyR1 particles did not have the opposing T‐tubule, so the presence of these modes of movements in our analysis suggests that the vertical elongation may also have additional functions.
*Mode 5*: (36% of variance compared to the mode 1): an outward twist of the cytoplasmic domains associated with an increase in membrane curvature ([Link], [Link]).

### Activation of RyR1 in native membranes

**Figure 4:**
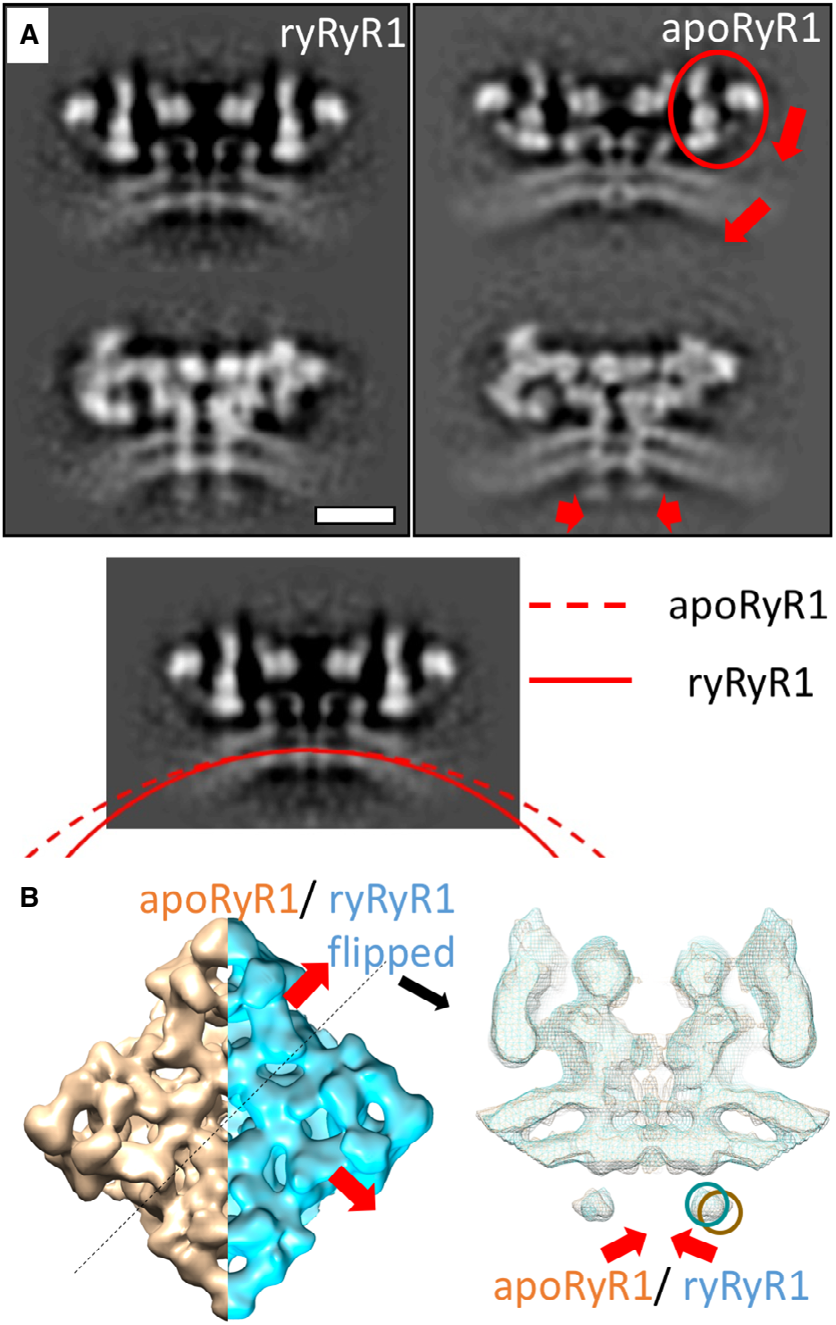
Activation of RyR1 *in situ* Structure of ryRyR1 at a resolution of 17.5 Å. Two views are through the middle slice (top) of the reconstruction and at the level of sarcoplasmic extensions 22 Å below (bottom panels). The movement of the outer domains is associated with a visible change in membrane curvature as compared to the apo structure. This movement is highlighted by red arrows in the right panels. Scale bar: 10 nm.A volume‐rendered representation of the apoRyR1 (orange) and ryRyR1 (blue) structures with the highlighted conformational changes. The ryRyR1 reconstruction was mirrored in the right panel for a clear comparison to the apoRyR1. The left panel depicts the view down onto the receptor from the cytoplasm. The right panel depicts the section through the isosurface indicated by the dashed line, with red arrows indicating the conformational transition of the SR extensions (dark orange and blue circles for apoRyR1 and ryRyR1, respectively). Red arrows indicate the conformational changes of the SR extensions.

**Figure EV4:**
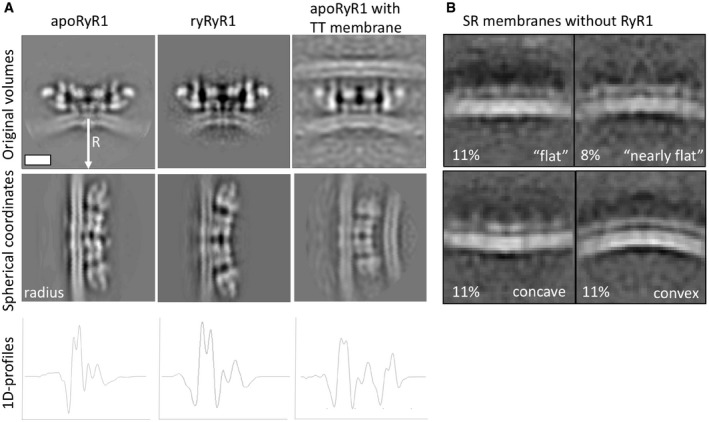
Measuring the local curvature of the membrane by transforming the density maps into spherical coordinates varying the distance to the origin of the coordinate system Maps of apoRyR1, ryRyR1 and apoRyR1 with the presumably TT membrane attached (top). Middle slice: the maps from the top row transformed into spherical coordinates carrying the distance “R” to the centre or the spherical transformation. The radius R was varied in order to optimize the flatness of the SR membrane in spherical coordinates, and the values are 50 nm for apoRyR1, 35 nm for ryRyR1 and 55 nm for apoRyR1‐TT.Most populated average patches of SR membranes without RyR1s in the presence of EDTA. Patches show different local curvature including concave, flat or near flat and convex. Four selected classes are shown with the numbers indicating the percentages of observations. Scale bar for both images: 10 nm.

We further analysed the structure of RyR1 in the presence of 0.3 mM Ca^2+^ and 10 μM ryanodine, which have previously been reported to lock the receptor in an open state 28. The structure was determined by cryo‐electron tomography and StA of 890 particles and resulted in a structure with a global resolution of 17.5 Å (Fig 4A and Appendix Fig S4). When compared to apoRyR1, the structure of RyR1 in the presence of Ca^2+^ and ryanodine, which we refer to as ryRyR1, shows movement of the outer domains of RyR1 down towards the SR membrane (Fig 4A and B, Movie EV7). In the previously reported single‐particle structures of purified receptor 16, 27, 28, similar conformational changes were suggested to be associated with the opening of the ion channel, suggesting that the captured conformation in our *in situ* structure corresponds to the open state of RyR1. Interestingly, we also observe a difference in the curvature of the SR membrane between apoRyR1 and ryRyR1, resulting in an additional displacement of the intra‐SR extensions inwards by approximately 1 nm (Fig 4B). The membrane curvature measured in the same section through RyR1 changes from approximately 1/50 nm^−1^ in the apo state to approximately 1/35 nm^−1^ (Figs 4A and EV4, and Movie EV8) in the presence of Ca^2+^ and ryanodine. The curvature of apoRyR1 attached to putative T‐tubule membrane had a curvature of 1/55 nm^−1^, slightly lower than that of apoRyR1 (Fig EV4). In comparison, patches of the SR membrane lacking RyR1 showed a wide range of curvatures from concave to convex, with the most common appearance near flat (Fig EV4). Mode 5 of the principal component analysis also shows a relationship between the conformation of the receptor and the local membrane curvature; however, it should be noted that the change in curvature is smaller than that observed upon the activation of the receptor.

The curved membrane surrounding RyR1 has been previously observed by Renken *et al* 23. The curvature observed in our structures is higher than the curvature in the subtomogram average structure reported by Renken *et al* 23, but was not as pronounced as the curvature seen in some views of the membrane around junctional RyR1s from native fish muscle 22. In general, membrane curvature can be established through various mechanisms, including (asymmetric) lipid composition or insertion of wedge‐shaped membrane proteins and/or amphipathic helices 42, 43. At the current resolution of our *in situ* structure, we were unable to identify any proteins asymmetrically inserted into the bilayer that could contribute to membrane curvature other than RyR1. It has previously been reported that SR membranes purified from rabbit muscle contain a significant proportion of phosphatidylethanolamine (PE) lipids (21% of total lipid contents), which can induce spontaneous curvature, and that 69% of PE lipids resided in the outer leaflet, while 85% of phosphatidylserine lipids and 88% of phosphatidylinositol lipids resided in the inner leaflet 44. The formation of the observed curvature *in vivo* is therefore likely to be a combination of lipid composition, asymmetric lipid distribution and the impact of multiple RyR1s clustered in close proximity to each other at the junctional SR.

Interestingly, the activation of the receptor increases the observed membrane curvature, a change that was not observed during a previous structural analysis of purified RyR1 reconstituted in nanodiscs 27. Fluctuations in membrane curvature in the absence of RyR1 activation are small and have only been observed in mode 5 of our PCA (Appendix Fig S4) and in 10% of the particles assigned to class 4, which had decreased membrane curvature (Appendix Fig S3). This suggests that the even higher membrane curvature observed in the ryRyR1 structure is likely a consequence of RyR1 activation. An increase in membrane curvature has two potential implications for the gating of RyR1. First, energy stored upon RyR1 opening in the deformed membrane can contribute to additional force in the direction of channel closing. As this force contributes to the energy landscape that defines the thermodynamic properties of channel opening and closing, it should be accounted for when performing computational modelling of the channel activation. Second, potential energy stored in the curved membrane could be used for mechanical regulation of neighbouring proteins located in the same membrane. Increased membrane curvature could increase the lateral pressure of lipids in the outer bilayer. SERCA, postulated based on size and shape to be the highly abundant SR membrane protein in our tomograms, occupies a larger area in the outer leaflet than it does in the inner leaflet when in an outward‐facing conformation 45, 46, also potentially leading to lateral pressure on the surrounding lipids. As SERCA and RyR1 cover most of the SR membrane surface, the open and closed states of each protein have the potential to manipulate membrane tension in opposing directions and therefore cross‐regulate each other.

Understanding conformational dynamics *in situ*, including interactions with binding partners and the native lipids, promises to be a comprehensive way to analyse the structure and function of proteins like RyR1 with similarly extensive regulation. For this, StA structures of RyR1 *in situ* still require higher resolution in order to better understand the molecular composition and regulation of the entire RyR1 interactome in native membranes. The current class averages reported from our data do not allow interpretation at the level of secondary structure due to resolution limitations. The resolution of our structures is limited by both the number of particles and sample heterogeneity: in the apoRyR1 reconstructions, the outer domains show significant lower resolution suggesting lower level of local order. In the case of RyR1 attached to putative T‐tubules, the number of particles and the order of the DHPR‐RyR1 interaction are potentially limiting factors preventing the observation of the DHPR‐RyR1 complex. Use of quicker data collection schemes 47, 48 currently in development may make it possible to record sufficiently large datasets in the future, which would allow better classification of the RyR1 conformational states *in situ* while still retaining a sufficient number of particles in each class to reach subnanometer resolution. Such structural analysis could be further complemented by the application of molecular dynamics simulations or manifold analysis [preprint: 30, 49]. High‐resolution classification *in situ* would allow better understanding of the interactions of RyR1 with its associated proteins, as well as quantitative understanding of the conformational landscape of the receptor activation in native membranes.

## Materials and Methods

### Sample preparation for cryo‐EM

SR vesicles were isolated based on the previously described protocol 32. Briefly, 60 g of fresh rabbit skeletal muscle tissue from the hind leg and back was ground using a meat grinder and then homogenized using a blender with 300 ml of homogenization buffer (0.5 mM EDTA, 10% sucrose, 20 mM Na_4_O_7_P_2_, 20 mM NaH_2_PO_4_ and 1 mM MgCl_2_, pH 7.1) plus the following protease inhibitors: 2.6 μg/ml aprotinin, 1.4 μg/ml pepstatin and 10 μg/ml leupeptin. Homogenates derived from a total of 180 g of muscle were centrifuged in a Beckman Coulter rotor JLA‐16.250 fixed‐angle rotor at 8,900 × *g* at 4°C for 20 min. The resulting supernatant was filtered through cheesecloth and then ultra‐centrifuged in a Beckman Coulter Type 45Ti fixed‐angle rotor at a speed of 20,000 × *g* at 4°C for 1 h. The membrane pellets were divided into 20 aliquots. One aliquot was used immediately in the next step, and the remaining aliquots were stored at −80°C for future use. The membrane pellet fraction was subjected to a discontinuous sucrose gradient with steps of 0.15 ml 50%, 1.27 ml 36%, 1.27 ml 34%, 1.58 ml 32%, 1.58 ml 28%, 3.8 ml 25% and 1.27 ml 14% sucrose. The sucrose gradient was then centrifuged in a Beckman Coulter SW 40Ti swinging‐bucket rotor at 96,200 × *g* for 90 min. Bands at the interface of the 25 and 28% sucrose phases and at the interface of the 28 and 32% sucrose phases were confirmed to contain RyR1 by Western blot. These bands were extracted from the sucrose gradient, diluted with dilution buffer (0.5 mM EDTA, 20 mM Na_4_O_7_P_2_, 20 mM NaH_2_PO_4_ and 1 mM MgCl_2_, pH 7.1) to 4 ml and then ultra‐centrifuged in a Beckman Coulter TLA 100.4 fixed‐angle rotor at a speed of 40,000 × *g* at 4°C for 20 min. The final membrane pellet was resuspended with 1 ml of dilution buffer. For apoRyR1, this suspension was used directly for cryo‐EM grid preparation. For ryRyR1, this suspension was dialysed against dialysis buffer (20 mM sodium pyrophosphate, 20 mM NaH_2_PO_4_, pH7.1) overnight at 4°C, and then Ca^2+^ was added to the sample to a final concentration of 0.3 mM Ca^2+^. The mixture was incubated at room temperature for 20 min before ryanodine was added to a final concentration of 10 μM. This final mixture was incubated overnight at 4°C and then used for cryo‐EM grid preparation. Male white New Zealand rabbits 11–12 weeks old from the Frankfurt University Medical School or Charles River Laboratories (https://www.criver.com/) were used. Three independent preparations have been performed for the EDTA dataset and one for the ryanodine dataset.

### Cryo‐EM grid preparation and tomographic data collection

Grids were frozen for cryo‐EM using a Vitrobot™ Mark IV (Thermo Fisher). 3 μl of the sample mixed with 10‐nm colloidal gold fiducials was applied to a 300‐mesh gold Quantifoil^®^ R 2/2 grid with gold support. The grid was blotted with Whatman^®^ No. 1 filter paper and plunged into liquid ethane cooled to liquid nitrogen temperature. Imaging was performed on a Thermo Fisher Titan Krios operated at 300 kV equipped with a Gatan K2 Summit^®^ direct electron detector and a Gatan Quantum^®^ energy filter. Single‐axis tilt series (−60° to +60°) were collected using a dose‐symmetric tilt‐scheme 50 with 3° intervals implemented in SerialEM 51. For some tomograms, the electron dose for the untilted image was increased to 18 e^−^/Å^2^, with the remaining projections receiving a dose of 1.1 e^−^/Å^2^ recorded in five frames; the total exposure was about 68 e^−^/Å^2^. Images were recorded at a magnification of 53,000×, resulting in a pixel size of 2.7 Å/pixel. Nominal defocus was set between 1.5 and 6 μm.

### Image processing

Per‐tilt motion correction was performed using MotionCor2 52, and defocus estimation was performed using Gctf for each projection 53. The tomographic tilt series were aligned using the 10‐nm gold fiducials in IMOD 54. Tomographic reconstructions were generated by weighted back‐projection implemented in IMOD 54. Nonlinear anisotropic diffusion filtering 55 was performed to aid particle picking in binned tomograms and for the figures. Subtomogram positions were picked manually with IMOD and extracted with a box size of 200^3^ cubic voxels from unbinned CTF‐corrected tomograms using the *dtcrop* function in Dynamo 56. Initial alignment was done manually with the *dynamo_gallery* followed by constrained refinement of shifts and angles using the Dynamo alignment *dcp* workflow. C4 symmetry was applied. Initial classification by multireference alignment was used to remove bad particles, after which independent half‐set refinement was performed for the datasets containing over 500 particles as previously described 57. For the datasets containing less than 501 particles, the frequency ranges of the resulting reconstructions were restricted to 36 Å which was much lower than the final resolution values. The final reconstructions were produced by taking into consideration dose‐dependent resolution decay for each particle, and the contribution of each of the particle to the final average was proportional to the particle's cross‐correlation to a reference. The resolution was determined by *dynamo_fsc*; local resolution and locally filtered maps were generated using Relion 2.0 58. Summary of the produced maps may be found in Appendix Table S1.

### Analysis of conformational heterogeneity

We used *Dynamo* to perform multireference alignment and classification of our final dataset into fifty classes using frequencies up to 22 Å and with applied C4 symmetry. From these fifty classes, we excluded fourteen classes due to low particle abundance or due to their inclusion of other confounding features, such as gold beads. We aligned the remaining 36 classes, containing a total of 2,105 particles, to a common average and performed eigenvolume analysis by principal component analysis (PCA). By this step, all 36 volumes were fully sampled in Fourier space and therefore no missing wedge needed to be accounted for. The half‐maps for each mode were generated by dividing these 36 averages into two equal‐sized groups according to their eigencoefficients, and these modes are presented in Fig 3D, Appendix Fig S3 and Movies EV2, EV3 , EV4 , EV5 , EV6 and EV7. The first principal component was trivial; therefore, the reported modes (1–5) are enumerated starting from the second principal component. The informativity of the classes presented in Fig 3E is the covariance along the corresponding eigenvector. This graph is the result of the PCA procedure implemented in *dynamo_ccmartix_analyze* and is presented in arbitrary units with the covariance of the first eigenvector set to 1.

Transmembrane segment prediction was performed using TMHMM v.2.0c 38.

## Author contributions

MK designed the research. WC performed the sample preparation and collected data. WC performed structural analysis with input from MK. MK and WC wrote the manuscript.

## Conflict of interest

The authors declare that they have no conflict of interest.

## Supporting information



Appendix

Expanded View Figures PDF

Movie EV1

Movie EV2

Movie EV3

Movie EV4

Movie EV5

Movie EV6

Movie EV7

Movie EV8

Review Process File

## Data Availability

Ten generated maps were deposited to EMDB with accession numbers EMD-10637, EMD-10638, EMD-10639, EMD-10640, EMD-10641, EMD-10642, EMD-10643, EMD-10644, EMD-10645 and EMD-10646. Original tilt series, particles and corresponding metadata are available under EMPIAR‐10349 (https://www.ebi.ac.uk/pdbe/emdb/empiar/entry/10349/).
