# Supplementary material for: Structure of RyR1 in native membranes
Source: EMBO Rep. 2020 Mar 9;21(5):e49891. doi: 10.15252/embr.201949891 (PMC7202208; doi:10.15252/embr.201949891)
Supplement: Supplementary file 1 — Appendix [file EMBR-21-e49891-s001.pdf]

**Appendix for**

# Structure of RyR1 in native membranes

Wenbo Chen, Mikhail Kudryashev

**Table of contents:**

Appendix Table S1 1
Appendix Figure S1. Structural similarities between the *in situ* structure and the previously reported high-resolution structures of purified RyR1s 2
Appendix Figure S2. Classification of apoRyR1 particles into four classes 3
Appendix Figure S3. Modes of structural variation of RyR1 *in situ* 4
Appendix Figure S4. Relation of spatial position and eigencoefficients from the structural modes presented in Fig 4 5
Appendix Figure S5. Structure of ryRyR1 6

## Appendix Table S1. Statistics of data collection and data processing

| | **EGTA / ApoRyR1, EMD-XXX** | **EGTA/apoRyR1-TT, EMD-XXX** | **ryRyR1, EMD-XXX** | **EDTA Class 1** | **EDTA Class 2** | **EDTA Class 3** | **EDTA Class 4** | **DDM+EDTA** | **DDM+1mM Ca2+** | **DDM+5mM Ca2+** |
|---|---|---|---|---|---|---|---|---|---|---|
| Microscope | Titan Krios | Titan Krios | Titan Krios | Titan Krios | Titan Krios | Titan Krios | Titan Krios | Titan Krios | Titan Krios | Titan Krios |
| Voltage, Cs | 300 / 2.7 | 300 / 2.7 | 300 / 2.7 | 300 / 2.7 | 300 / 2.7 | 300 / 2.7 | 300 / 2.7 | 300 / 2.7 | 300 / 2.7 | 300 / 2.7 |
| Camera | Gatan K2 with a quantum GIF | Gatan K2 with a quantum GIF | Gatan K2 with a quantum GIF | Gatan K2 with a quantum GIF | Gatan K2 with a quantum GIF | Gatan K2 with a quantum GIF | Gatan K2 with a quantum GIF | Gatan K2 with a quantum GIF | Gatan K2 with a quantum GIF | Gatan K2 with a quantum GIF |
| Pixel Size | 2.7 Å | 2.7 Å | 2.7 Å | 2.7 Å | 2.7 Å | 2.7 Å | 2.7 Å | 2.7 Å | 2.7 Å | 2.7 Å |
| Total electron dose e-/A2 | 68 | 68 | 68 | 68 | 68 | 68 | 68 | 68 | 68 | 68 |
| Defocus range, um | 4-5 um | 4-5 um | 1.5-5 um | 4-5 um | 4-5 um | 4-5 um | 4-5 um | 4-5 um | 4-5 um | 4-5 um |
| Number of tomograms used | 82 | 82 | 47 | 82 | 82 | 82 | 82 | 10 | 9 | 10 |
| Number of particles in the final map | 2547 | 205 | 890 | 1138 | 679 | 478 | 252 | 243 | 301 | 140 |
| Symmetry of the map | C4 | C4 | C4 | C4 | C4 | C4 | C4 | C4 | C4 | C4 |
| Refinement method | independent half-set | non-independent | independent half-set | non-independent | non-independent | non-independent | non-independent | non-independent | non-independent | non-independent |
| Resolution, Å | 12.6 / 12...15.5 | 38 at fsc = 0.5 | 17.5 | 15.4 at fsc=0.3 | 18 at fsc=0.3 | 18 at fsc=0.3 | 21.6 at fsc=0.5 | 25.7 at fsc = 0.5 | 27.7 at fsc = 0.5 | 30 at fsc = 0.5 |
| Applied B-factor | -1200 | 0 | 0 | 0 | 0 | 0 | 0 | 0 | 0 | 0 |

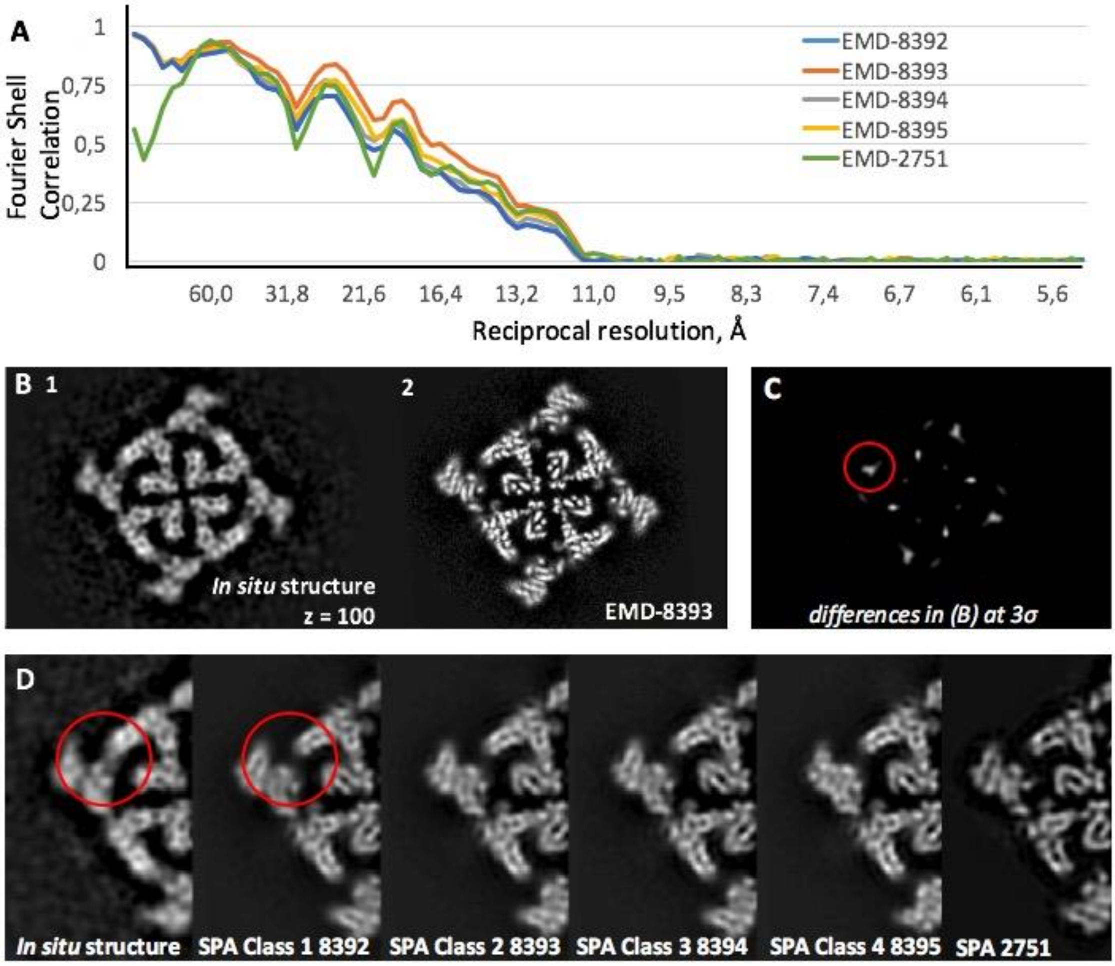


**Appendix Figure S1. Structural similarities between the *in situ* structure and the previously reported high-resolution structures of purified RyRs.**
A. Fourier Shell Correlation between the mutually aligned *in situ* structure and the respective reconstructions of purified receptors by single particle cryo-EM [27, 28].
B-D. Panels showing slices through the *in situ* structure and the reported single particle cryo-EM structures of the purified receptors filtered to 15 Å for comparison. Panel C is a difference between the volumes in panels B1 and B2 at a contrast level of 3*σ.

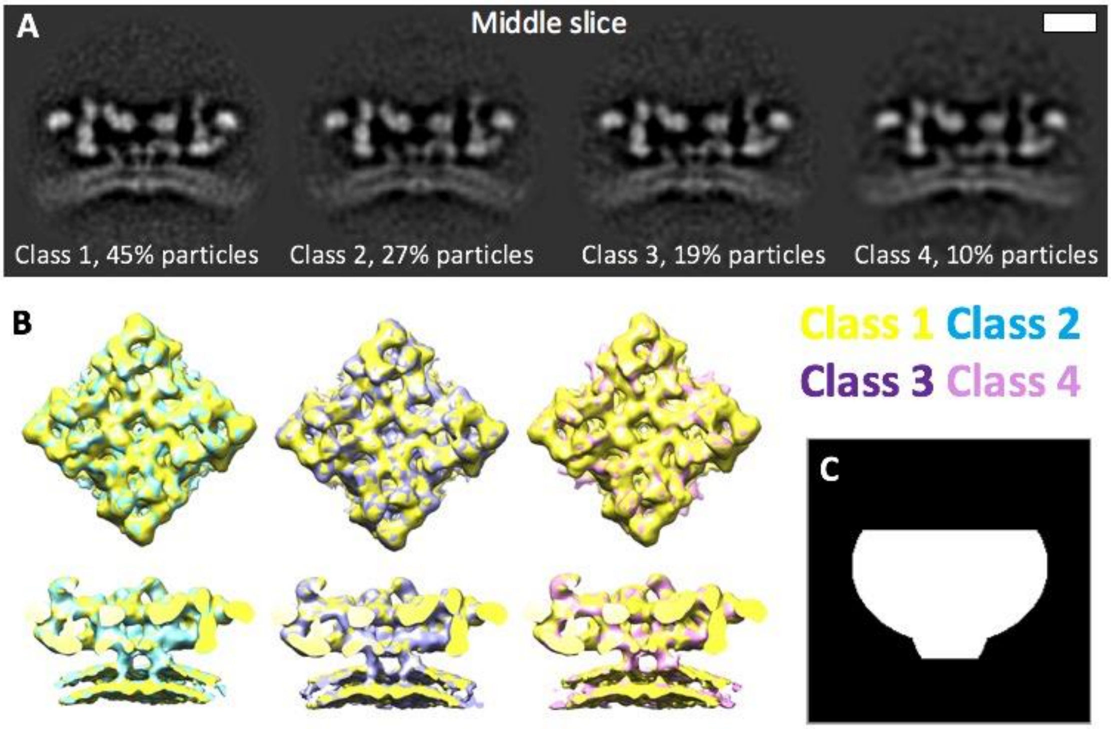


**Appendix Figure S2. Classification of apoRyR1 particles into four classes.**
A. Slices thought the class averages at 15.4, 18, 18 and 27 Å respectively. Scale bar: 10 nm.
B. Isosurface representation of the differences between the classes viewing from the cytoplasmic side and in the central section through the volume-rendered representation.
C. A mask used for multi-reference alignment and classification.

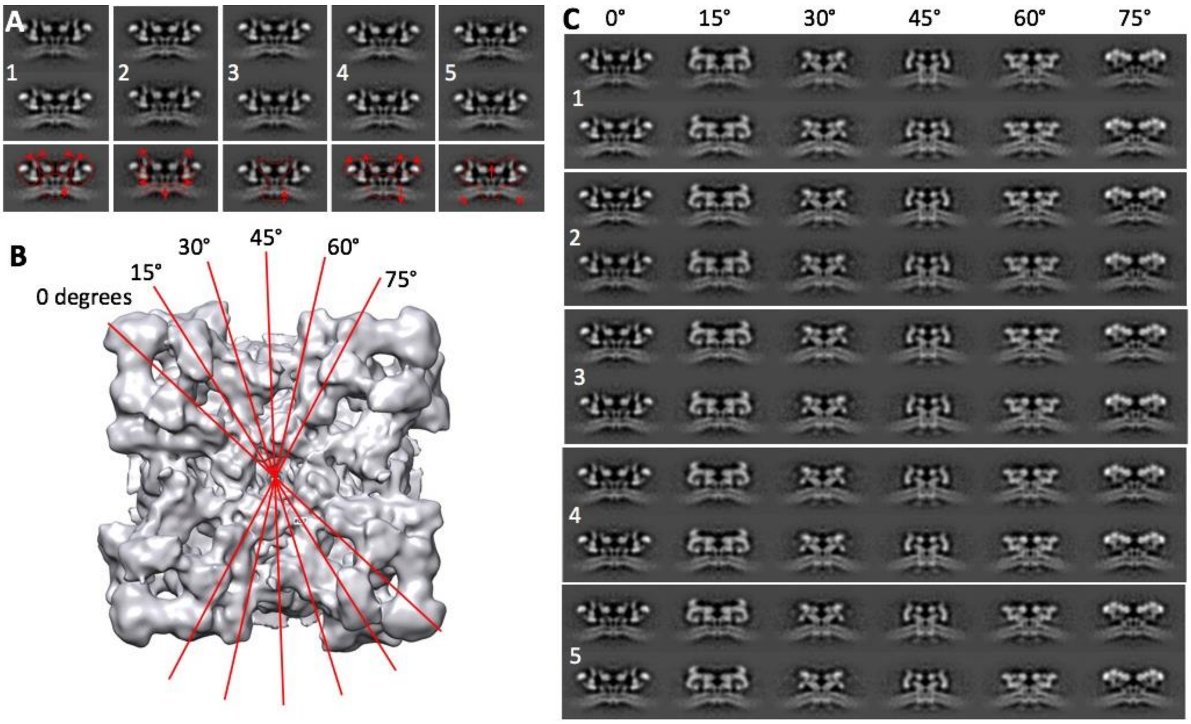


**Appendix Figure S3. Modes of structural variation of RyR1 *in situ*.**

A. Transitions between half-sets of each class summed according to their distribution in the first five modes.

B. The schematic at bottom-left shows the sections separated at 15° steps that are displayed in the panels at right. Movie EV2 toggles between the states in the top-left while movies EV3-EV7 demonstrate conformational modes from all the views.

C. The slices through the half-sets divided according to the modes as in (A) in six different directions with 15 degrees' step. The panels are the same as in movies EV3-EV7.

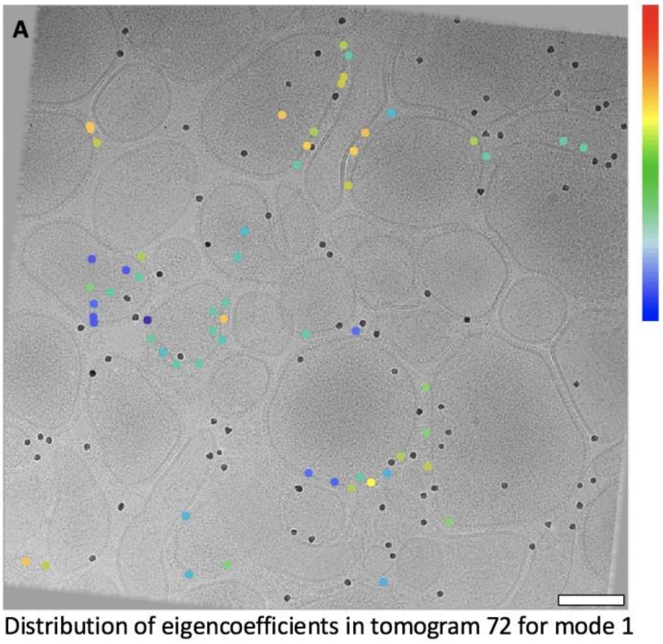


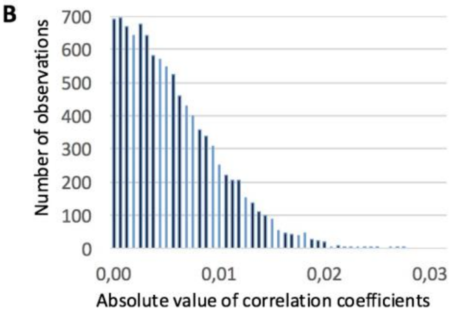


C

| Mode | CC | p-value |
|---|---|---|
| 1 | 0,0244 | **0,0003** |
| 2 | 0,0004 | 0,95 |
| 3 | 0,0016 | 0,82 |
| 4 | 0,0126 | 0,07 |
| 5 | 0,0007 | 0,92 |
| 6 | 0,0042 | 0,55 |
| 7 | 0,0012 | 0,86 |
| 8 | 0,0135 | 0,05 |
| 9 | 0,0134 | 0,05 |

**Appendix Figure S4. Relation of spatial position and eigencoefficients from the structural modes presented in Fig 4.**

A. Locations of multiple RyR1s overlaid on the cryo-EM micrograph color-coded according to the eigencoeffieients related to mode 1. Scale bar: 100 nm.

B. Distribution of correlation coefficients for particle-to-particle distances to a random variable with n=84062 measurements for 10 000 random initializations of the random variables.

C. A table with cross-correlation values between the distance between the particles and the difference between their eigencoefficients along the listed modes. P-values are derived from the simulations presented in panel B.

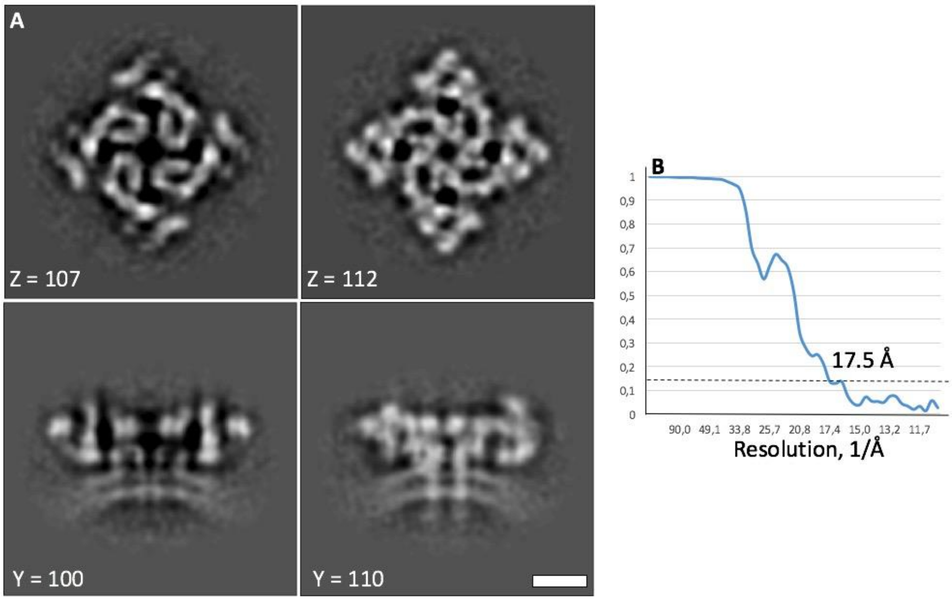


**Appendix Figure S5. Structure of ryRyR1.**

A. Slices through the structure with the locations of the sections indicated by the Y or Z coordinates. Scale bar: 10 nm.

B. Fourier Shell Correlation between independently refined half-sets indicates a resolution of the reconstruction of 17.5 Å.
