# Supplementary material for: Structure of RyR1 in native membranes
Source: EMBO Rep. 2020 Mar 9;21(5):e49891. doi: 10.15252/embr.201949891 (PMC7202208; doi:10.15252/embr.201949891)
Supplement: Supplementary file 2 — Expanded View Figures PDF [file EMBR-21-e49891-s002.pdf]

Expanded View Figures

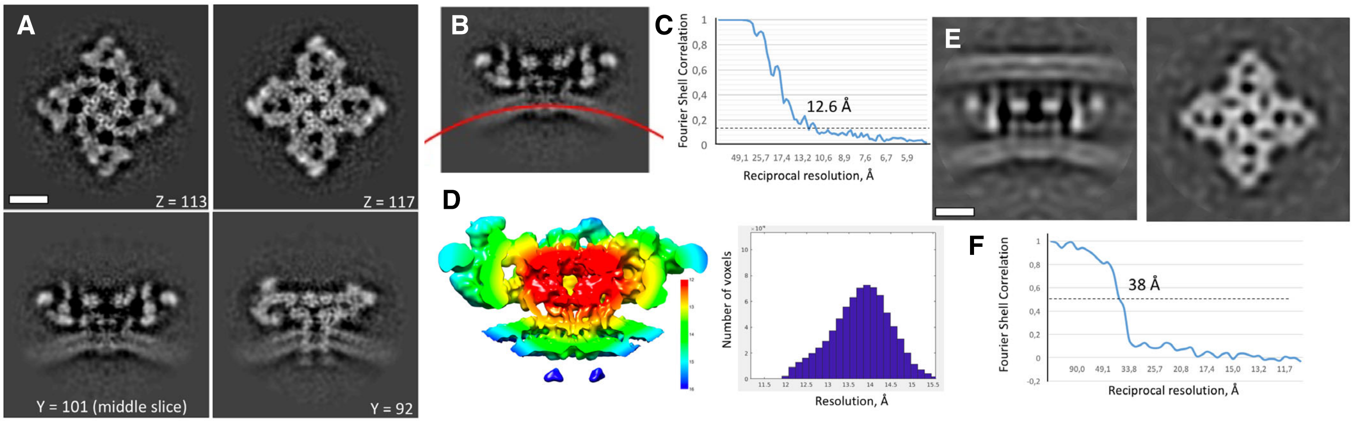

Figure EV1. Structure of apoRyR1 *in situ*.

A Slices through the *in situ* average map or apoRyR1 in XY and XZ directions. Scale bar: 10 nm. Please note that due to observation through different section, the apparent curvature in the same structure may change. Therefore, it is critical to compare the curvature either at the same section or in sections traversing the middle of the particle.

B Measurement of the membrane curvature in the average structure.

C Resolution measurement of the structure in apo state by Fourier shell correlation between independently processed half-maps.

D Local resolution of the structure with the corresponding colour code.

E, F Structure of apoRyR1 in contact with the putative T-tubule membrane and the corresponding resolution curve. Scale bars: 10 nm.

 © 2020 The Authors

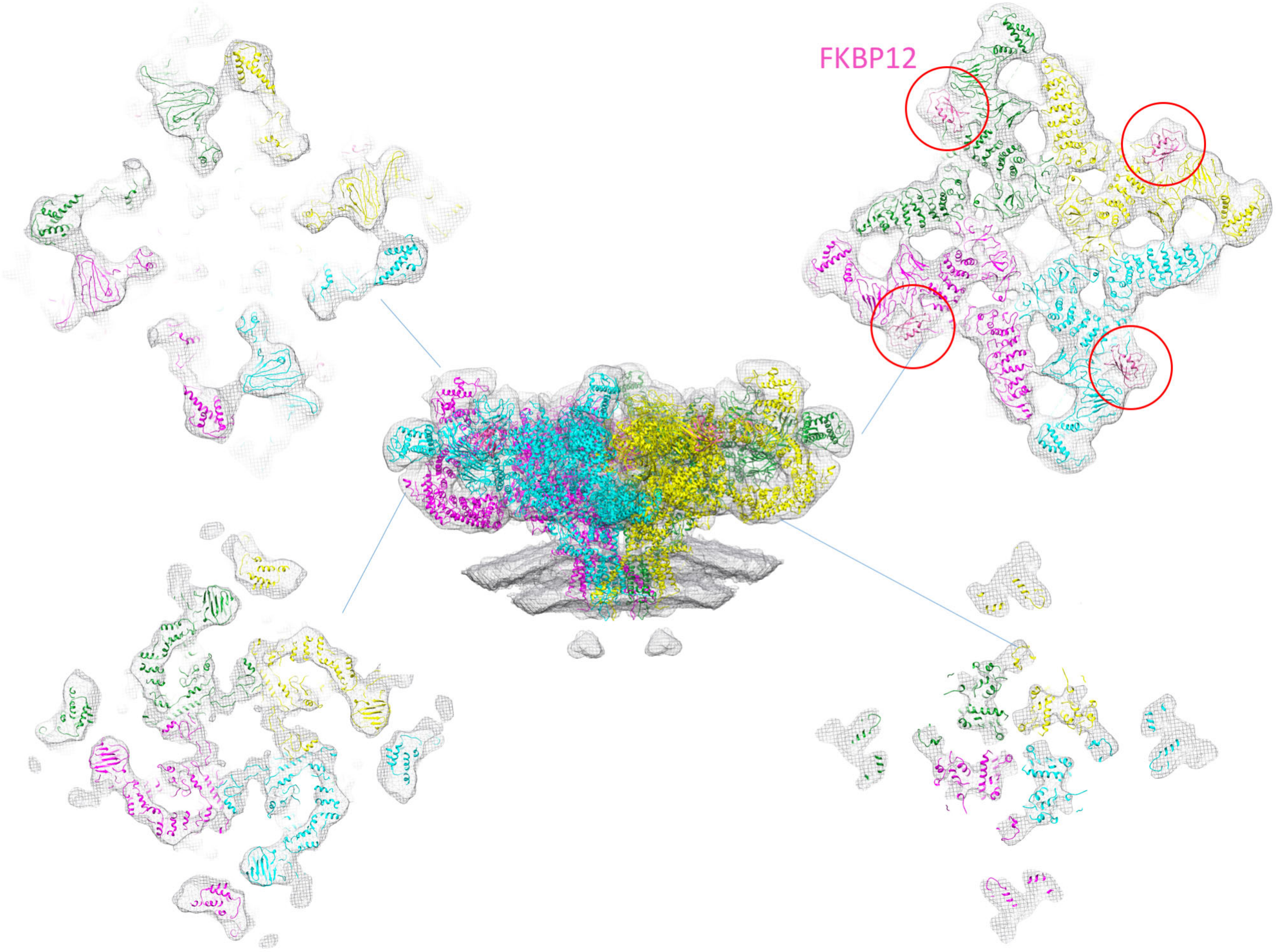

Figure EV2. Rigid-body docking of the atomic model 5TB2 to the *in situ* structure.

Slices through isosurface are presented perpendicular to the central panel at the heights indicated by the blue lines. FKBP12 is marked by red circles on the top right panel.

© 2020 The Authors

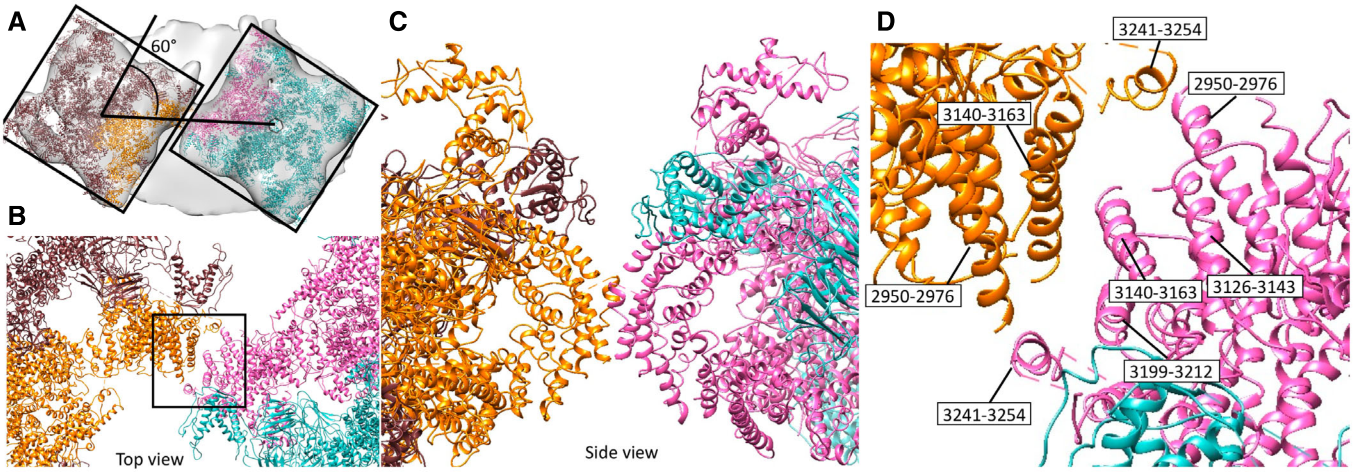

Figure EV3. Inter-protomer interactions between apoRyR1s in the paracrystalline array in native membranes.

A A subtomogram average map including two neighbouring apoRyR1s with two atomic models 5TB2 fitted.

B, C Top and side views on the atomic models of the interacting domains.

D Zoom in to the area shown in (B) with the labelled interacting helices.

 © 2020 The Authors

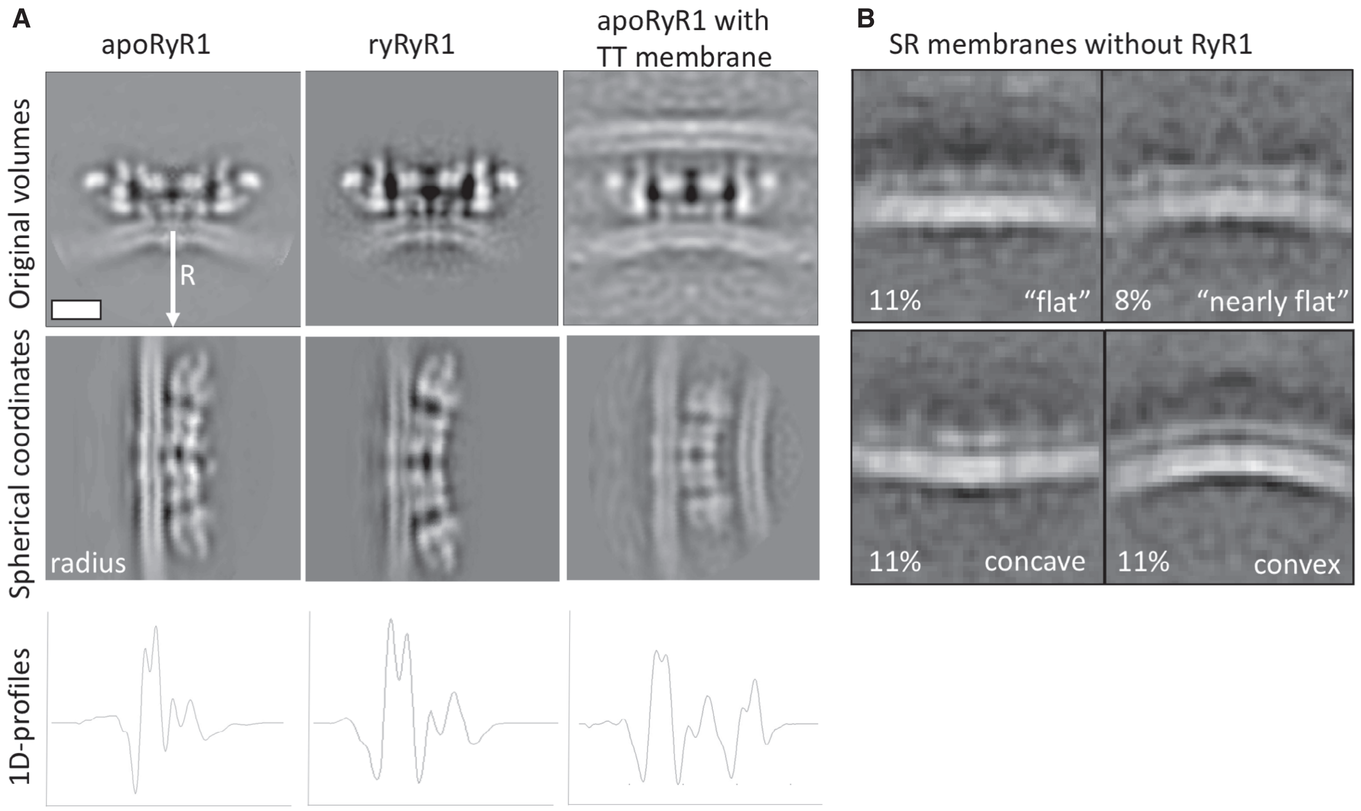

Figure EV4. Measuring the local curvature of the membrane by transforming the density maps into spherical coordinates varying the distance to the origin of the coordinate system.

A Maps of apoRyR1, ryRyR1 and apoRyR1 with the presumably TT membrane attached (top). Middle slice: the maps from the top row transformed into spherical coordinates carrying the distance "R" to the centre or the spherical transformation. The radius R was varied in order to optimize the flatness of the SR membrane in spherical coordinates, and the values are 50 nm for apoRyR1, 35 nm for ryRyR1 and 55 nm for apoRyR1-TT.

B Most populated average patches of SR membranes without RyR1s in the presence of EDTA. Patches show different local curvature including concave, flat or near flat and convex. Four selected classes are shown with the numbers indicating the percentages of observations. Scale bar for both images: 10 nm.

© 2020 The Authors
